# Approaching the Ecological Role of the Squat Lobster (*Munida gregaria*) and the Fuegian Sprat (*Sprattus fuegensis*) in the Francisco Coloane Marine Area (Magellan Strait, Chile) Using a Pelagic Food Web Model

**DOI:** 10.3390/ani13010003

**Published:** 2022-12-20

**Authors:** Daniela Haro, Sergio Neira, Juan Carlos Hernández-Padilla, Francisco Arreguín-Sánchez, Pablo Sabat, Cristian Vargas

**Affiliations:** 1 Centro Bahía Lomas, Facultad de Ciencias, Universidad Santo Tomas, Ignacio Carrera Pinto 1350, Punta Arenas 6200000, Chile; 2 Center for Oceanographic Research COPAS COASTAL ANID FB210021, Departamento de Oceanografía, Universidad de Concepción, Víctor Lamas St. 1290, Concepción 4030000, Chile; 3 Centro de Investigación y de Estudios Avanzados del Instituto Politécnico, CINVESTAV, Unidad Mérida Km. 6 Antigua Carretera a Progreso Apdo. Postal 73, Mérida 97310, Mexico; 4 Instituto Politécnico Nacional, Centro Interdisciplinario de Ciencias Marinas, P.O. Box 592, La Paz 23090, Mexico; 5 Laboratorio de Ecofisiología, Facultad de Ciencias, Universidad de Chile, Las Palmeras 3425, Ñuñoa, Santiago 7750000, Chile; 6 Center of Applied Ecology and Sustainability (CAPES), Pontificia Universidad Católica de Chile, Avenida Libertador Bernardo O’Higgins 340, Santiago 7500945, Chile; 7 Instituto de Fomento Pesquero, Enrique Abello 680, Punta Arenas 6200000, Chile

**Keywords:** trophic role, ecosystem model, structure and functioning, squat lobster, Fuegian sprat, Magellan Strait

## Abstract

**Simple Summary:**

We investigated the ecological role of the squat lobster (*Munida gregaria*) and the Fuegian sprat (*Sprattus fuegensis*) in the food web of the Francisco Coloane Marine Area in the Magellan Strait, Chile. We analyzed the ecosystem impacts of biomass changes in the squat lobster and Fuegian sprat. Food web indicators and simulations were estimated using the Ecopath with Ecosim software. Both species were located in the second trophic level. The squat lobster was preyed upon by 12 functional groups, which highlighted its role as an important prey in the ecosystem and its positive impacts on predators such as red cod, whales, and penguins. As prey, the Fuegian sprat presented a direct interaction with 10 functional groups, which exerted positive impacts on predators such as penguins, seabirds and whales. In summary, the Francisco Coloane Area is an immature ecosystem with productivity and energy flows values within those reported for productive ecosystems; the role of the squat lobster seems to be related to the structure of the food web, while the Fuegian sprat plays a role in the energy transfer to top predators. Although the study area is currently a Marine Protected Area, monitoring squat lobster and Fuegian sprat populations might ensure the conservation of this ecosystem.

**Abstract:**

The structure and functioning of the food web of the Francisco Coloane Marine Area in the Magellan Strait, Chile, was quantified, with an emphasis on identifying the ecological role of the squat lobster (*Munida gregaria*) and the Fuegian sprat (*Sprattus fuegensis*). Food web indicators, the trophic level, and centrality indices were estimated using Ecopath with Ecosim. Dynamic simulations were carried out to evaluate the ecosystem impacts of biomass changes in squat lobster and Fuegian sprat. The model calculated a total ecosystem biomass of 71.7 t km^−2^ and a total primary production of 2450.9 t km^−2^ year^−1^. Squat lobster and Fuegian sprat were located in specific trophic levels of 2.3 and 2.7, respectively. Squat lobster reduction produced a decrease in the biomass of red cod (42–56%) and humpback whales (25–28%) and Fuegian sprat reduction a decrease in penguins (15–37%) and seabirds (11–34%). The Francisco Coloane Area is an immature ecosystem with productivity and energy flows values within those reported for productive ecosystems; the role of the squat lobster seems to be related to the structure of the food web, and the role of the Fuegian sprat seems to be related to the functioning of the ecosystem and to the energy transfer to top predators.

## 1. Introduction

The first attempts at food web analysis in aquatic and terrestrial ecosystems focused on the study of energy flow patterns and ecosystem structure [1,2,3]. Most of the subsequent studies focused on determining the control mechanisms of energy flow by analyzing the importance of resource limitation (bottom-up control) and predation (top-down control) in the structure of the ecosystem [3,4,5]. The study of the role played by the various organisms that make up the food web acquired great interest (e.g., [6,7,8,9]). It has been shown that the organisms that make up the zooplankton have a pelagic–benthic role. These organisms transport organic matter to the benthic community from the pelagic realm through their feces and their vertical migration. These groups also play a role in the food web structure by transferring energy to higher trophic levels [8,10,11]. Small pelagic fish may exert a top-down control on their zooplankton prey and at the same time a bottom-up control on their fish, bird, and marine mammal predators [12,13]. Therefore, small pelagic fish control the system trophic dynamics by exerting a “wasp waist” control [12,13,14], which has been reported in various highly productive ecosystems such as the Humboldt, Canarias, and Benguela currents [12].

In recent decades, the role of a functional group and its potential to fulfill a key species function has been analyzed in different ecosystems using trophic models with mass balance through the Ecopath with Ecosim (EwE) approach (e.g., [7,15,16]). These models quantify food web structure and energy flow in aquatic ecosystems [17,18] and also allow identification of the ecological role of each functional group, calculation of network indicators, and assessment of the ecosystem effects of changes in group biomass [19].

The system of fjords and channels in the Magellan region of southern Chile is an extensive geographical area (8500 km of coastline) that is composed of coastal and oceanic water masses [20]. The oceanographic conditions are determined by complex marine–terrestrial–atmospheric interactions that result in unique marine ecosystems with high biological production [21,22,23]. In the Magallanes region, the squat lobsters *Munida gregaria* and *M. surugosa* are the dominant group in terms of biomass [24,25]. Due to their abundance; their importance as prey for various predators such as cephalopods, fish, birds, and marine mammals [26]; and their direct connection between lower and higher trophic levels, *Munida* spp. have been suggested as key species in the coastal ecosystems of southern South America [10,27,28]. The small pelagic Fuegian sprat (*Sprattus fuegensis*), a species that is present in high biomass during spring and autumn months [29,30], is a fishing resource species in the northern area of the fjords and channels [31,32]. The Fuegian sprat is prey for the large predators (penguins, seabirds, sea lions, and whales) present in the area [33] and for fishery species such as the southern hake (*Merluccius australis*), long-tailed hake (*Macruronus magellanicus*), and pink cusk-eel (*Genypterus blacodes*) [34]. This suggests that this species could play key ecosystem roles both as a predator and as prey [32].

The Francisco Coloane Marine Area in the Magellan Strait, which has a pelagic ecosystem with a high diversity of fauna, has recorded at least 14 species of marine mammals and 32 marine and/or coastal birds [35,36,37]; it is also a feeding area for Magellanic penguins (*Spheniscus magellanicus*), humpback whales (*Megaptera novaeangliae*), and sea lions [38]. Although these large predator populations are supported by species such as the squat lobster and Fuegian sprat [33,34,39,40], no information exists regarding the ecological role these crustaceans and fish play in the ecosystem and the impact that possible biomass changes in these species would have on the food web. According to the role previously indicated for *Munida* spp. in southern coastal ecosystems and its importance in the diet of various species [26,27,33,40], we hypothesized that the squat lobster fulfills a role in connecting basal trophic level groups and higher trophic level groups in the study area. In accordance with previous reports on small pelagic fish in productive ecosystems and the fact that the Fuegian sprat has a role as both predator and prey in the fjord and channel system, we also hypothesized that the Fuegian sprat plays a role related to energy transfer in the Francisco Coloane ecosystem. The objective of this study was to analyze quantitatively the food web structure of the Francisco Coloane Marine Area in the Magellan Strait with an emphasis on the ecological roles played by the squat lobster and the Fuegian sprat through a trophic model using Ecopath with Ecosim.

## 2. Materials and Methods

**Figure 1:**
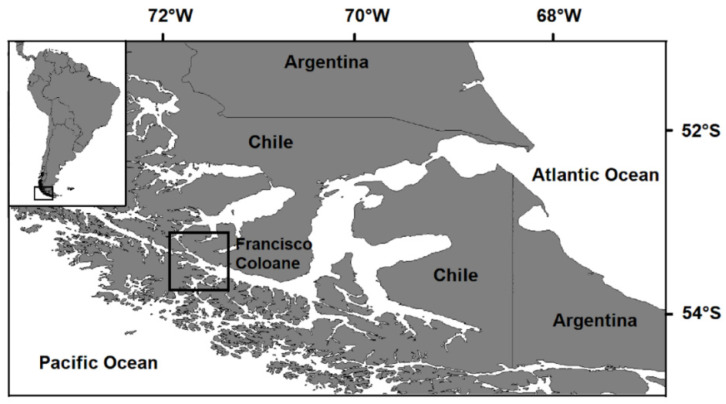
Study area in Chile: Francisco Coloane Marine and Coastal Protected Area in the Magellan Strait, Chile (square).

The Francisco Coloane Marine and Coastal Protected Area is located in the central section of the Magellan Strait (53°38′ S, 72°14′ W, Figure 1); it has an approximate surface area of 670 km^2^. The creation of this area aids the conservation of feeding areas for the humpback whale and breeding areas for the Magellanic penguin and the common sea lion (*Otaria byronia*) [35]. The previously published Ecopath with Ecosim model of the Francisco Coloane Marine Area [41] was used to characterize the food web structure in that model, and scenarios were examined for the simulation. This model represents the period 2007 to 2017 and includes 21 functional groups corresponding to the main trophic functional groups of the system. The model considered three groups of marine mammals, two groups of birds, eight groups of fish, five groups of invertebrates, one group representing benthic organisms, one group for the phytoplankton, and one group of detritus (Table 1). More details about the model are presented in [41].

### 2.1. Ecosystem Structure

The network and food web indicators that described the ecosystem properties and energy flows proposed in [42] were estimated by using routines included in the Ecopath with Ecosim software that used the total flow index, which is a measure of the size of the system in terms of flows, and Finn’s cycling index for the total flow fraction used for recycling [43]. The Ecopath with Ecosim model was used to calculate the trophic level (TL) of each functional group as a fractional number by assigning a trophic level of =1 to primary producers and detritus and a trophic level of 1+ to consumers [44] with the following equation:

(1) $$TL_{j}=1+\overset{n}{\underset{i=1}{\sum }}DC_{ji}\cdot TL_{i}$$

where $\overset{n}{\underset{i=1}{\sum }}DC_{ji}$ is the average of the prey consumed by *j* and $TL_{i}$ is the trophic level of prey *i*. To analyze the distribution of biomass and energy flux at each trophic level, the functional groups were aggregated into a simple food web with five discrete levels as proposed in [45].

### 2.2. Ecological Role of Munida gregaria and Sprattus fuegensis

*Munida gregaria* is a species with two morphotypes: ‘gregaria’ (pelagic) and ‘subrugosa’ (benthic) [46]. As the constructed model focused on the pelagic zone of the study area, the squat lobster was considered the ‘gregarious’ morphotype. The analysis of the direct and indirect trophic interactions was carried out using mixed trophic impacts (MTIs), which is a routine in Ecopath with Ecosim that analyzes the effects that a biomass change in one functional group could have on the biomass of other groups in the system [47]. The MTIs were calculated through a matrix of proportions with fluctuating values between −1 and 1, which represented the proportional change of each group *i* in the event of an increase in the group that impacted *j*. The matrix was calculated using the following equation:

(2) $$MTI_{ji}=DC_{ji}-FC_{ji}$$

where $DC_{ji}$ is the fraction of *i* in the diet of *j* and $FC_{ji}$ is the proportion of predation on *i* due to predator *j*.

The predator–prey consumption matrix of the Ecopath model (Table 2) was used to estimate the centrality indices, which describe the structural characteristics of the food web and allow the determination of the key species in a given system [16,48,49]. The indices used included the degree index (*D_i_*), which quantifies the number of connections between groups and expresses how they connect to the rest of the food web [50] according to:

(3) $$D_{i}=D_{in,i}+D_{out,i}$$

where $D_{i}$ is the degree of functional group *i*, $D_{in,i}$ is the number of connections between a consumer and its prey, and $D_{out,i}$ is the number of connections between a group *i* and its predators. The closeness index (*CC_i_*) quantifies the number of connections or flow paths between a group and all other groups in the system [51]; i.e., it measures how close a group is to the other groups in the system. This index was calculated using the equation proposed in [52]:

(4) $$CC_{i}=\frac{1}{\sum_{j=1}^{n}d_{ij}}$$

where *i* ≠ *j* and $d_{ij}$ is the shortest path distance between groups *i* and *j* in the food web. The intermediation index (*BC_i_*) measures positional importance based on how frequently a functional group *i* is on the shortest path between every pair of groups *j* and *k* and provides an approximation of the importance of a group as a connector within the system. This index was estimated as:

(5) $$BC_{i}=\underset{j<k}{\sum }\frac{g_{jki}}{g_{jk}}$$

where *i* ≠ *j*, *k*, $g_{jki}$ is the number of short routes in which a group *i* is incident, and $g_{jk}$ is the lowest number of trophic routes between groups *j* and *k*. All centrality indices were estimated using the Visone 2.6.3 software.

### 2.3. Ecosim Simulations 

Ecosim, a model that uses the temporal dynamics of EwE, estimates biomass changes in each functional group through the differential equations of the Ecopath model using the temporal variation in catch rates and biomass [19,45]. The expression of the Ecosim differential equation is:

(6) $$\frac{dBi}{dt}=f\left(B\right)-M_{O}B_{i}-F_{i}B_{i}-\overset{n}{\underset{j=1}{\sum }}C_{ij}\left(B_{i},B_{j}\right)$$

where f(B) is a function of *B_i_* and expresses the production of *i*, $M_{O}$ is the mortality rate of group *i* due to causes other than predation and capture, *B_i_* is the biomass of group *i*, *F_i_* is the fishing mortality of *i*, and *C_ij_* ($B_{i},B_{j}$) is function that predicts the consumption of prey *i* by predator *j*. Dynamic simulations were carried out in Ecosim to analyze the roles of the squat lobster and the Fuegian sprat in the structure and distribution of energy flow in the ecosystem. Three fishing extraction scenarios were simulated for squat lobster and three for Fuegian sprat that included the removal of 10%, 25% and 50% of the biomass of each. The extraction of both species was also simulated by removing 25% of their biomass. All simulations were run for 30 years. In all simulations, a constant fishing extraction of 0.5 t km^2^ of squat lobster or Fuegian sprat was used to analyze the effect of these changes on the relative biomass of the other functional groups in the system.

## 3. Results

### 3.1. Ecosystem Structure 

The Ecopath model indicated that the total ecosystem biomass (excluding detritus) was 71.7 t km^−2^, while the total primary production reached 2450.9 t km^−2^ year^−1^. The total system flows were 5796.9 t km^−2^ year^−1^ including consumption, exports, respiration, and flows to detritus in the ecosystem (Table 3). Finn’s cycling index was 1.6 and the mean of food chains was 2.4.

**Figure 2:**
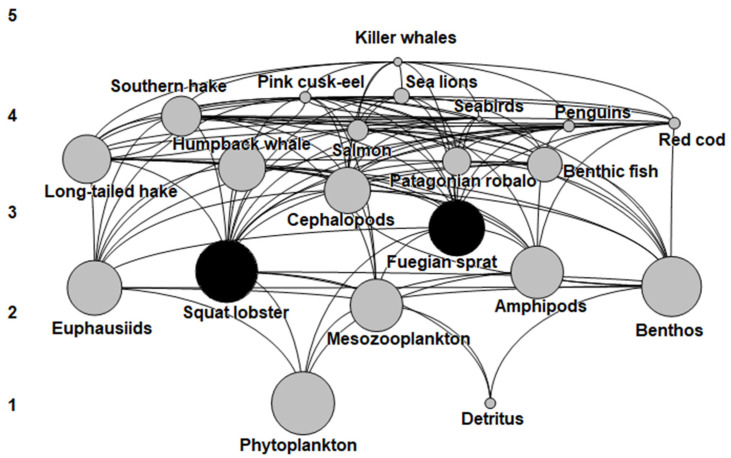
Ecosystem flow diagram of the Francisco Coloane Marine Area. Groups are distributed on the vertical axis according to their trophic level (i.e., 1–5). The circles are proportional to the functional biomass size of the groups. Squat lobster and Fuegian sprat groups are in black.

The trophic level of the functional groups varied from 1.0 for primary producers (phytoplankton) and detritus to 4.4 for top predators such as killer whales. Squat lobster and Fuegian sprat were in the second trophic level and presented levels of 2.3 and 2.7, respectively (Figure 2, Table 4). A large proportion of matter in fractional trophic level II flowed through mesozooplankton (1.0 t km year^−1^), euphausiids (0.8 t km year^−1^), and benthos (0.8 t km year^−1^); and in trophic level III through Fuegian sprat (0.7 t km year^−1^) and cephalopods (0.8 t km year^−1^). Matter flows in squat lobster were higher at trophic level II. The flows of large predators such as penguins (0.6 t km year^−1^) and killer whales (0.5 t km year^−1^) stood out in the upper trophic levels (Table 5).

### 3.2. Analysis of the Ecosystem Role of Squat Lobster and Fuegian Sprat

**Figure 3:**
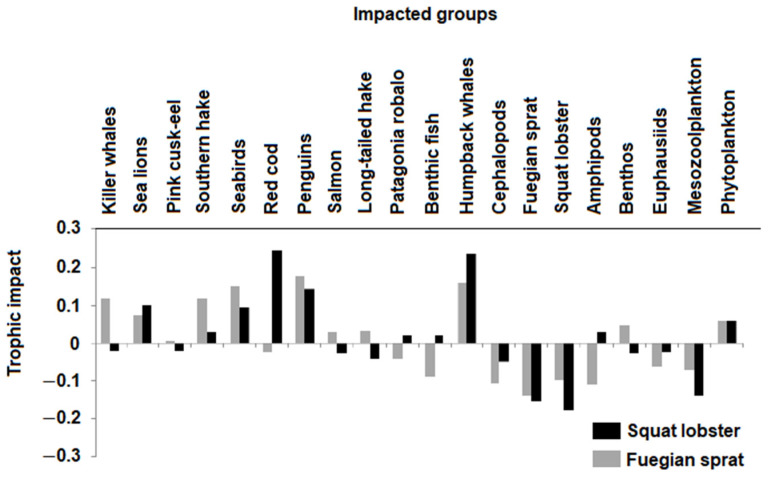
Trophic impacts exerted by the squat lobster (black bar) and the Fuegian sprat (gray bar) on the other functional groups of the Francisco Coloane ecosystem, Magellan Strait.

The Ecopath model indicated that the squat lobster was preyed upon by 12 functional groups in the system, especially red cod, humpback whales, and penguins. As a predator, the squat lobster was related to low trophic level groups such as detritus, phytoplankton, and mesozooplankton (Figure 2, Table 2). The squat lobster exerted a positive impact of 20% on red cod and humpback whales, a positive impact of 10% on penguins, and negative impacts on competing groups such as the Fuegian sprat (20%) and prey groups such as mesozooplankton (10%) (Figure 3). When the Fuegian sprat was considered as prey, it presented a direct relationship with 10 functional groups including penguins, seabirds, humpback whales, and sea lions. As a predator, the Fuegian sprat was related to zooplankton and phytoplankton groups (Figure 2, Table 2). Trophic impacts indicated that the Fuegian sprat had a positive impact of 20% on penguins, humpback whales, and birds; a positive impact of 10% on killer whales and southern hake; and a negative impact of 10% on competing groups such as cephalopods and squat lobsters (Figure 3). Killer whales, southern hake, and squat lobster showed the highest values for the degree index (D*_i_*). The CC*_i_* index was high in killer whales, squat lobster, Fuegian sprat, and southern hake; killer whales had the highest value in the BC*_i_* index followed by squat lobster (Table 4).

### 3.3. Ecosim Simulations

**Figure 4:**
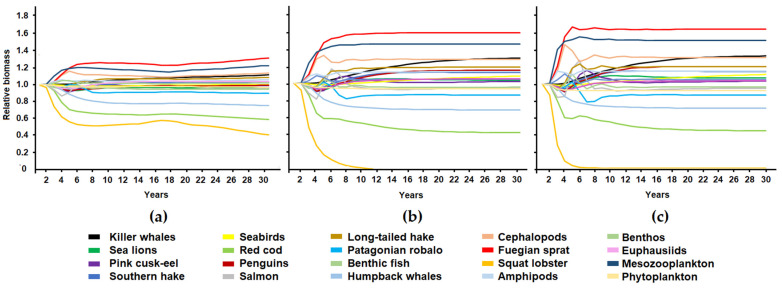
Changes in the relative biomass of the functional groups of the ecosystem in three simulated scenarios for squat lobster showing fishing extraction of 10 (**a**), 25 (**b**), and 50% (**c**) of its biomass.

**Figure 5:**
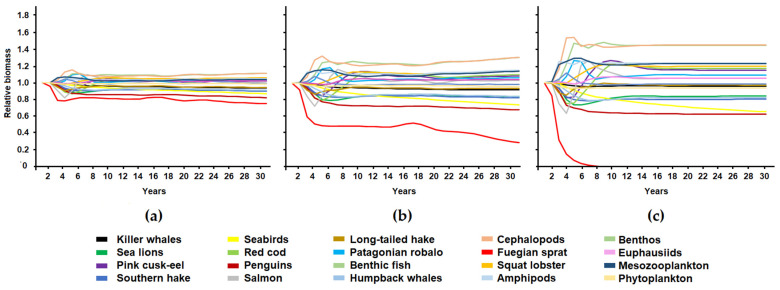
Changes in the relative biomass of the functional groups of the ecosystem in three simulated scenarios for Fuegian sprat showing fishing extraction of 10 (**a**), 25 (**b**), and 50% (**c**) of its biomass.

Figure 4 and Figure 5 show the changes in the relative biomass of functional groups in the scenarios that simulated different catch levels for the squat lobster and Fuegian sprat. Changes in the functional groups’ biomass over time followed the same trend in the three scenarios; however, there was difference in the magnitude of these changes. Removal of 10% of the squat lobster biomass produced fluctuations of up to 40% of the biomass of the red cod and squat lobster; in contrast, the removal of 50% of the squat lobster caused variations of up to 65% of the relative biomass of the functional groups of the system (Fuegian sprat) and the collapse of its own population (Figure 4). The simulation of the squat lobster fishery produced an increase in the biomass of Fuegian sprat (between 31 and 69% depending on the fishing scenario) and mesozooplankton (22–57%), a decrease in groups of fish such as red cod (42–56%) and Patagonian robalo (11–21%), and a decrease in humpback whales (25–28%). Sea lions, euphausiids, and benthos maintained their biomass without substantial changes (Figure 4).

Removal of 10% of the Fuegian sprat’s biomass produced changes of less than 20% of the biomass of the groups in the system, while the removal of 50% caused fluctuations close to 50% in the relative biomass of some functional groups (cephalopods and benthic fish) and the collapse of its own population (Figure 5). The fishing removal of Fuegian sprat resulted in a biomass increase in cephalopods (10–53%), benthic fish (10–47%), mesozooplankton (1–30%), and amphipods (1–25%); and a decline in the biomass of penguins (15–37%), seabirds (11–34%), sea lions (10–26%), humpback whales (9–21%), and southern hake (9–21%). Benthic organisms, phytoplankton, long-tailed hake, and killer whales did not show significant changes in their relative biomass (Figure 5).

**Figure 6:**
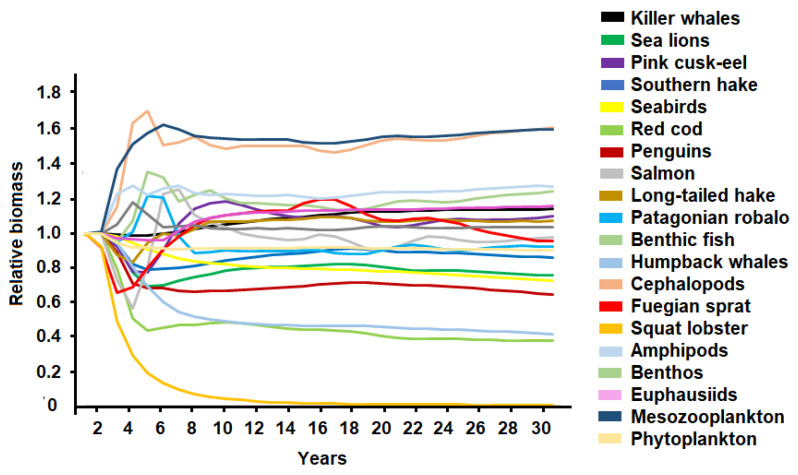
Changes in the relative biomass of the functional groups of the ecosystem when simulating the fishing extraction of 25% of the biomass of squat lobster and Fuegian sprat.

The removal of squat lobster and Fuegian sprat together produced an average increase of 54% in the relative biomass of mesozooplankton and 52% in the biomass of cephalopods (Figure 6). It also caused a decrease in the relative biomass of red cod (54%), humpback whales (48%), penguins (30%), seabirds (19%), sea lions (21%), and southern hake (13%).

## 4. Discussion

### 4.1. Ecosystem Structure

Several models have been developed using the EwE approach to study the structure of different food webs (e.g., [53,54]). In the fjords and channels of southern Chile, these models have been applied mainly to the inner Sea of Chiloé and northern Patagonia (41°–46° S) to analyze the food web structure and primary productivity [55,56] as well as the ecological role of the Fuegian sprat [32] and the South American sea lion [57]. Arancibia et al. 2010 [58] modeled the food web of the Magallanes region by considering an area comprising the interior and exterior waters to analyze the ecosystem impacts of the demersal fishery operating in the area. The model used in this study was the first trophic model with EwE in inland waters of the Magellan Strait and the first in the Francisco Coloane Marine Protected Area.

The total biomass (71.7 t km^−2^) in the study area was lower than values reported for other productive systems such as the pelagic ecosystem of northern Chile (707 t km^−2^ [59]), the system of Peru (225 t km^−2^ [60]), the west coast of Greenland (158 t km^−2^ [61]), and the Gulf of Saint Lawrence in the northern Atlantic (319 t km^−2^ [62]). The total primary production (2450.9 t km^2^ year^−1^) and total flow (5796.9 t km year^−1^) were lower than the productivity and flow reported in northern Chile [59] and an upwelling in Peru [60]. However, they were higher on the west coast of Greenland [61] and in the Gulf of St. Lawrence [62]. The trophic models built using the EwE approach can be different according to the biomass of the groups and the units of each parameter, so it is necessary to be careful when comparing among models. In addition, our study area was rather small (670 km^2^) compared to the areas of other published models such as the 35,059 km^2^ zone in the ecosystem of northern Chile [59] or the 103,812 km^2^ area in St. Lawrence in the North Atlantic [62], which likely influenced the lower values of total biomass found. Even so, the productivity and energy flow values found were within those reported for ecosystems with high productivity and chlorophyll-a concentration as described in the Marine Area [23,63,64].

One way to make relative comparisons between different ecosystems is to use the ratio (production/total system flow) as an indicator between two ecosystem parameters [65]. The SP/FT (sum of all production/total flows of the system) and PP/BT (total primary production/total biomass) (Table 3) ratios were higher than those reported in tropical coastal ecosystems [65,66,67] and in high-latitude ecosystems [68], but they were lower than those reported in the upwelling system of northern Chile [69]. These results indicated that the Francisco Coloane system presented a high production rate and a large amount of energy flowing through the ecosystem. The PP/RT (total primary production/total respiration) ratio, Finn’s cycling index, and the food web length are parameters used to determine the degree of system maturity [70]; there should be values greater than 1.0 for the PP/RT ratio and low recycling values in systems far from maturity [71]. Upwelling ecosystems are an example of immature systems that depend on energy rushing through them, thereby rendering them unstable and vulnerable to changes in nutrient input [72]. This study showed that the PP/RT ratio was higher than 1.0, which indicated an immature system. At the same time, the energy cycling was rather low (1.6%) compared to other systems that showed cycling higher than 20% [72,73]. It was close to the recycling reported in the ecosystem of upwelling of northern Chile (2.8%) and the average of 2.4 food chains found and the mean of 2.34 described for the system in northern Chile [59]. These indicators confirmed that the study area was a system far from maturity and were similar to those that occur in upwelling systems.

The fractional trophic levels ranged from 1.0 for primary producers to 4.4 for top predators; squat lobster and Fuegian sprat were at the second trophic level (Table 4). These values were within those reported for species such as the squat lobster found in the second trophic level [57] and for small pelagic fish located at a mean trophic level of 2.6 [7,32,74]. In the second fractional trophic level, the relative flows of euphausiids and benthic organisms stood out; in the third level, it was those of the Fuegian sprat and cephalopods. This highlighted the importance of these groups for energy flow in this food web as lower- and upper-level connectors, which coincided with reports for euphausiids [6,8] and pelagic fish such as the Fuegian sprat [7,14,16] in food web energy transfer.

### 4.2. Trophic Role of Munida gregaria and Sprattus fuegensis 

Trophic impacts of the squat lobster were slightly higher (0.05 units) than those of the Fuegian sprat; both groups exerted positive impacts on large predators and negative impacts on lower trophic levels. The model results demonstrated the relevance of the squat lobster and Fuegian sprat in trophic connections as prey to 60% (squat lobster) and 50% (Fuegian sprat) of the system groups. Several indices have been proposed to determine the potential of an organism to be a keystone species or to play a critical role in a community [15,48], such as the degree (D*_i_*) and centrality (CC*_i_* and BC*_i_*) indices used to analyze the functions of different organisms when considering the trophic interactions of the group under study as prey and predator in addition to the size of the food web (e.g., [9,16,49,75,76]). In this study, killer whales and the squat lobster exhibited high values in all these indices; the southern hake had high values in the D*_i_* and CC*_i_* indices as did the Fuegian sprat in the CC*_i_* index. These results indicated that the squat lobster has a role as a connector of the food web components [50,51] just like the southern hake. The Fuegian sprat also showed a high value in the CC*_i_* index, which indicated its importance in the energy flow. Similar results were reported by Hernández-Padilla et al. 2017 in the Gulf of California, where organisms from lower trophic levels and higher predators presented high values in the centrality indices, which suggested a bottom-up control energy flow due to many links in the low trophic levels [77].

### 4.3. Ecosim Simulations

The simulations carried out with the Ecosim model demonstrated the impact that a squat lobster or Fuegian sprat fishery could have on the relative biomass of other functional groups in the Francisco Coloane Marine Area in the Magellan Strait. Under fishing scenarios, depending on the amount of fishing, both populations could collapse in less than 10 years. Fishing removal of squat lobster caused a decline in the relative biomass of red cod, Patagonian robalo, and humpback whales but an increase in Fuegian sprat and mesozooplankton, and these trends were maintained over time. These results suggested the existence of competition between the squat lobster and the Fuegian sprat because when one of the populations decreases, the other increases. The squat lobster has a diet composed of detritus, phytoplankton, and zooplankton [10,27,78]; the Fuegian sprat feeds mainly on zooplankton and to a lesser extent on phytoplankton [41,79]. Therefore, the competition between both groups would be associated with trophic resources and highlight the negative effect exerted by both species on the relative abundance of zooplankton (Figure 3). These results agreed with Diez et al., 2018 [80], who reported spatial competition between *Munida gregaria* and *Sprattus fuegensis* associated with trophic resources in the Beagle Channel (54°–55° S). Fishing simulation results for the Fuegian sprat also show its importance for the higher trophic levels because the biomass of top predators declined (penguins, seabirds, sea lions, hake, and humpback whales). An abundance time series of the Fuegian sprat, its prey, and direct predators would be key information to demonstrate that in northern Chilean Patagonia, the Fuegian sprat plays a key role as a planktophagous predator and as prey for predators, thereby directly influencing the abundance of these groups, results that would be similar to this study finding. In upwelling ecosystems such as Peru, Benguela, and Monterrey, the exploitation of small pelagic fish caused an increase in the zooplankton biomass [81,82]. However, other studies indicated that small pelagic fish exploitation did not cause a significant biomass disturbance in groups of lower trophic levels [7,83], which suggested that the role exerted by small pelagic fish may vary in different ecosystems depending on the productivity and upwelling intensity in each system [7].

Due to its trophic interactions as both predator and prey, its high values in the degree and centrality indices, and its impact on the abundance of other species in the community, it was suggested that the squat lobster fulfills a function related to the connection of groups from lower trophic levels (detritus and phytoplankton) and higher trophic levels (humpback whales and red cod) in the food web. These results agreed with those indicated by Romero et al., 2004 and Lovrich and Thiel 2011 [84,85], who reported that *Munida gregaria* represents a direct link between particulate organic matter, primary producers, and top predators and is a key component of benthic–pelagic coupling [86]. Our results coincided with reports of highly connected species within a food web suggesting that the squat lobster plays a key role in food web structure according to its effect on the other component populations of the community [48,87]. Finally, the results on the relative fluxes and the CC*_i_* index strongly suggested that the Fuegian sprat is a significant species in the transfer of energy flux and functioning of the ecosystem. Although studies have reported that the squat lobster and Fuegian sprat play essential roles in the ecosystems they inhabit [88,89], the results of our study suggested that the role of the squat lobster seems to be related to the connection of the ecosystem functional groups. The role of the Fuegian sprat is related to the group connections and to energy flow transfer because they are larger organisms that consume a greater amount of biomass (and energy) and transfer it to top predators. 

In the event of a fishery for squat lobster and/or Fuegian sprat, it is suggested that the capture quotas of both resources be evaluated. It is also suggested that the implications that these fisheries would have on populations of higher predators be considered. Monitoring *Munida gregaria* and *Sprattus fuegensis* populations is suggested to ensure the protection and conservation of this ecosystem.

## 5. Conclusions

The food web in the Francisco Coloane Marine Area in the Magellan Strait is far from maturity and has productivity and energy flow values within those reported for productive ecosystems. The role of the squat lobster in this ecosystem seems to be related to the structure of the food web, and its potential fishery could affect mainly groups at intermediate/higher trophic levels. The role of the Fuegian sprat seems to be related to the functioning of the ecosystem and the energy transfer to top predators.

## Figures and Tables

**Table 1:** Basic input parameters and biomasses of the functional groups of the model in the Francisco Coloane Marine Area, Magellan Strait. B: biomass (t km^2^), P/B: production/biomass (year^−1^), Q/B: consumption/biomass (year^−1^), EE: ecotrophic efficiency, Y: fishing landing (t km^2^ year^−1^). Values estimated by the model are in bold.

Functional Group	Species	B	P/B	Q/B	EE	Y
Killer whales	*Orcinus orca*	0.065	0.118	7.760	**0.000**	-
Sea lions	*Otaria flavescens, Ar* *ctocephalus australis*	0.189	0.238	29.180	**0.259**	-
Pink cusk-eel	*Genypterus blacodes*	0.101	0.680	2.000	**0.964**	0.005
Southern hake	*Merluccius australis*	1.931	0.310	2.074	**0.952**	0.005
Seabirds	*Thalassarche melanophrys, Macronectes giganteus, Phalacrocorax atriceps, Larus dominicanus, Stercorarius chilensis, Fulmarus glacialoides, Ardenna grisea*	0.029	0.047	6.104	**0.410**	-
Red cod	*Salilota australis*	0.116	0.828	3.500	**0.886**	0.001
Penguins	*Spheniscus magellanicus*	0.100	0.240	58.230	**0.021**	-
Salmon	*Oncorhynchus tshawytscha, Salmo trutta, Oncorhynchus mykiss*	**0.353**	2.210	6.500	0.990	-
Long-tailed hake	*Macruronus magellanicus*	3.731	1.220	11.300	**0.449**	0.003
Patagonian robalo	*Eleginops maclovinus*	**0.709**	1.112	3.500	0.990	-
Benthic fish	*Patagonotothen cornucola, P. tessellata, Sebastes oculatus*	**1.194**	1.560	4.010	0.990	-
Humpback whale	*Megaptera novaeangliae*	3.367	0.139	7.011	**0.000**	-
Cephalopods	*Megalocyathus enteroctopus, Doryteuthis gahi*	**3.116**	3.500	12.800	0.990	-
Fuegian sprat	*Sprattus fuegensis*	**6.575**	3.000	9.251	0.990	-
Squat lobster	*Munida gregaria*	**11.506**	1.330	11.600	0.990	-
Amphipods	*Themisto gaudichaudii*	**5.055**	7.300	28.000	0.990	-
Benthos	*Pseudechinus magellanicus, Loxechinus albus, Lithodes santolla, Platynereis australis, Mytilus chilensis, Perumytilus purpuratus, Nacella magellanica, Kerguelenella lateralis, Fissurella radiosa, Margarella violácea*	**9.735**	2.700	11.640	0.990	0.290
Euphausiids	*Euphausia lucens, E. vallentini*	**6.308**	2.960	16.200	0.990	-
Mesozzoplankton		**5.096**	35.000	154.500	0.990	-
Phytoplankton		12.430	197.200	-	**0.381**	-

**Table 2:** Consumption matrix of functional groups of the Francisco Coloane Marine Area ecosystem. Consumptions of squat lobster and Fuegian sprats are shown in bold.

	Prey\Predador	1	2	3	4	5	6	7	8	9	10	11	12	13	14	15	16	17	18	19	21
1	Killer whales	-	-	-	-	-	-	-	-	-	-	-	-	-	-	-	-	-	-	-	0.109
2	Sea lions	0.012	-	-	-	-	-	-	-	-	-	-	-	-	-	-	-	-	-	-	1.137
3	Pink cusk-eel	0.005	0.052	-	-	0.000	-	-	-	0.004	-	-	-	-	-	-	-	-	-	-	0.043
4	Southern hake	0.111	0.056	0.025	-	0.021	0.036	0.052	-	0.263	-	-	-	-	-	-	-	-	-	-	0.829
5	Seabirds	0.001	-	-	-	0.000	-	-	-	-	-	-	-	-	-	-	-	-	-	-	0.036
6	Red cod	0.028	0.028	0.000	0.028	-	-	-	-	-	-	-	-	-	-	-	-	-	-	-	0.092
7	Penguins	0.001	-	-	-	-	-	-	-	-	-	-	-	-	-	-	-	-	-	-	1.188
8	Salmon	0.044	0.713	0.015	-	-	-	-	-	-	-	-	-	-	-	-	-	-	-	-	0.467
9	Long-tailed hake	0.111	0.768	0.025	1.047	0.021	0.033	-	-	0.037	-	-	-	-	-	-	-	-	-	-	1.094
10	Patagonian robalo	-	0.321	0.019	-	0.000	-	0.006	0.435	-	-	-	-	-	-	-	-	-	-	-	0.505
11	Benthic fish	-	0.518	0.029	0.445	-	0.073	0.027	0.199	0.042	0.511	-	-	-	-	-	-	-	-	-	0.976
12	Humpback whales	-	-	-	-	-	-	-	-	-	-	-	-	-	-	-	-	-	-	-	5.189
13	Cephalopods	0.129	0.768	0.011	0.364	0.021	0.018	1.947	0.319	5.636	-	0.939	-	0.294	-	-	-	0.351	-	-	8.085
14	Fuegian sprat	0.064	1.427	0.022	0.946	0.060	0.054	2.381	0.348	8.120	-	-	6.105	-	-	-	-	-	-	-	13.430
15	Squat lobster	-	0.865	0.019	0.475	0.031	0.148	1.409	0.272	2.775	0.184	0.469	7.869	0.634	-	-	-	-	-	-	31.950
16	Amphipods	-	-	0.007	-	-	0.000	-	-	8.767	-	1.408	2.931	11.740	**6.283**	-	-	5.901	-	-	29.080
17	Benthos	-	-	0.019	0.475	-	0.044	-	0.723	10.690	1.789	1.971	-	8.179	**-**	**0.159**	-	0.637	1.073	-	22.950
18	Euphausiids	-	-	0.000	0.222	0.021	0.000	-	-	5.821	-	-	6.701	4.491	**1.336**	**-**	-	-	-	-	20.740
19	Mesozooplankton	-	-	0.011	0.004	-	-	-	-	-	-	-	-	14.540	**42.520**	**53.940**	48.320	12.960	17.040	-	170.800
20	Phytoplankton	-	-	-	-	-	-	-	-	-	-	-	-	-	**15.940**	**52.360**	95.190	-	84.670	685.2	1518.000
21	Detritus	-	-	-	-	-	-	-	-	-	-	-	-	-	**-**	**52.360**	-	93.610	-	159.2	-
	Import	0	0	0	0	0	0	0	0	0	0	0	0	0	**0**	**0**	0	0	0	0	0
	Sum	0.504	5.516	0.202	4.005	0.177	0.406	5.823	2.296	42.160	2.485	4.787	23.610	39.878	**66.080**	**158.800**	143.500	113.500	102.780	844.300	1836.000

**Table 3:** Ecological indicators and estimated flow rates in the ecosystem of the Francisco Coloane Marine Area, Magellan Strait.

Parameter	Value
Sum of all consumption (t km^−2^ year^−1^)	1470.6
Sum of all the respiration flows (t km^−2^ year^−1^)	861.0
Sum of all flows to detritus (t km^−2^ year^−1^)	1875.4
Sum of all the exports (t km^−2^ year^−1^)	1589.8
Sum of all the production (t km^−2^ year^−1^)	2766.3
Total system flows (t km^−2^ year^−1^)	5796.9
Total biomass (without detritus) (t km^−2^)	71.7
Total primary production (t km^−2^ year^−1^)	2450.9
Finn’s cycling index (%)	1.6
Finn’s mean route length	2.4
Mean trophic level of the catch	2.3
Sum of all the production/total system flows (SP/FT)	0.5
Total primary production/total biomass (PP/BT)	34.2
Total primary production/total respiration (PP/RT)	3.8

**Table 4:** Trophic level and functional indices of each ecosystem group in the Francisco Coloane Marine Area ecosystem.

Functional Groups	Trophic Level	D*_i_*	CC*_i_*	BC*_i_*
Killer whales	4.49	1.09	1.00	0.13
Sea lions	4.13	0.64	0.69	0.00
Pink cusk-eel	4.12	0.68	0.71	0.00
Southern hake	3.93	0.91	0.85	0.02
Seabirds	3.91	0.59	0.71	0.01
Red cod	3.86	0.59	0.71	0.00
Penguins	3.83	0.59	0.71	0.01
Salmon	3.79	0.50	0.67	0.00
Long-tailed hake	3.49	0.68	0.76	0.01
Patagonian robalo	3.46	0.68	0.76	0.01
Benthic fish	3.44	0.64	0.69	0.01
Humpback whales	3.41	0.32	0.60	0.00
Cephalopods	3.17	0.77	0.82	0.02
Fuegian sprat	2.79	0.82	0.85	0.02
Squat lobster	2.34	0.86	0.88	0.04
Amphipods	2.34	0.64	0.73	0.01
Benthos	2.19	0.68	0.76	0.02
Euphausiids	2.18	0.64	0.73	0.01
Mesozooplankton	2.00	0.59	0.67	0.00
Phytoplankton	1.00	0.41	0.60	0.00

**Table 5:** Relative fluxes (t km year^−1^) at each discrete trophic level in the Francisco Coloane Marine Area ecosystem as proposed in [45].

Functional Groups	I	II	III	IV	V
Killer whales	-	-	-	0.5	0.3
Sea lions	-	-	0.1	0.5	0.2
Pink cusk-eel	-	-	0.2	0.4	0.2
Southern hake	-	-	0.2	0.5	0.1
Seabirds	-	-	0.2	0.5	0.1
Red cod	-	-	0.3	0.4	0.1
Penguins	-	-	0.2	0.6	-
Salmon	-	-	0.3	0.4	0.1
Long-tailed hake	-	-	0.5	0.4	-
Patagonian robalo	-	-	0.6	0.2	-
Benthic fish	-	-	0.6	0.3	-
Humpback whales	-	-	0.5	0.3	-
Cephalopods	-	-	0.8	0.1	-
Fuegian sprat	-	0.2	0.7	-	-
Squat lobster	-	0.6	0.3	-	-
Amphipods	-	0.6	0.3	-	-
Benthos	-	0.8	0.1	-	-
Euphausiids	-	0.8	0.1	-	-
Mesozooplankton	-	1.0	-	-	-
Phytoplankton	1.0	-	-	-	-
Detritus	1.0	-	-	-	-

## Data Availability

None of the data were deposited in an official repository. The data are available from the authors upon request.
